# The Impact of Unmet Healthcare Needs on the Perceived Health Status of Older Europeans During COVID-19

**DOI:** 10.3389/ijph.2024.1607336

**Published:** 2024-09-30

**Authors:** Šime Smolić, Nikola Blaževski, Margareta Fabijančić

**Affiliations:** Faculty of Economics and Business, University of Zagreb, Zagreb, Croatia

**Keywords:** unmet healthcare needs, older adults, SHARE Corona survey, COVID-19 exposure, Europe

## Abstract

**Objectives:**

To examine how unmet healthcare needs and the exposure to the pandemic impacted self-reported health (SRH) among individuals aged 50 and above.

**Methods:**

We use data from two waves of the Survey of Health, Ageing and Retirement in Europe (SHARE) Corona Survey collected in 2020 and 2021 in 27 European countries and Israel (*N* = 42,854). Three dimensions of barriers to healthcare access were investigated: healthcare forgone, postponed, and denied. Mixed-effects logistic regression analysis was employed to explore SRH deterioration during the pandemic.

**Results:**

Findings indicate that unmet healthcare needs decreased throughout the pandemic but significantly contributed to the worsening of SRH among older adults. Mild or severe exposure to the pandemic heightened the likelihood of reporting deteriorated SRH. Additionally, the pandemic disproportionately affected females, the oldest-old, and those living alone or facing economic vulnerability.

**Conclusion:**

To mitigate the adverse effects on the health status of older adults, policymakers are strongly advised to prioritize addressing the healthcare needs of those who have been disproportionately affected by the pandemic.

## Introduction

After the COVID-19 outbreak, healthcare systems introduced a range of approaches to curb the spread of the novel coronavirus [1]. Although these actions reduced the virus transmission rate, they also interrupted healthcare provisions [2–4]. Securing access to healthcare and acquiring essential health resources are crucial for preserving or improving health [5]. Nevertheless, when access is restricted, it can lead to severe and long-lasting health problems, reduce quality of life (QoL), worsen health status, and exacerbate health inequalities [6]. In addition to other consequences brought about by the pandemic, the restricted availability of healthcare services influenced the overall health status of those affected [7]. Adhering to home confinement and minimizing in-person interactions with others deteriorated the physical health of older adults, particularly those who remained isolated at home [8, 9]. Furthermore, the increase in physical inactivity during the COVID-19 lockdowns [10] and higher perception of the risk of infection and COVID-19-related death [11] have been linked with significant decreases in life satisfaction and both physical and mental health (MH).

While examining possible unintended consequences of the COVID-19 pandemic, French et al. [12] showed that the female sex and an increased concern about coronavirus infection were risk factors for physical and mental health status. Lüdecke and von dem Knesebeck [13] found that the deterioration in self-reported health (SRH) among older adults became more common as the pandemic extended across Europe and that “disparities in SRH were more prominent for individuals without a partner or with lower educational attainment and less pronounced for females and those aged 70+.” Similarly, Moens et al. [14] highlighted that women and older adults with poor SRH experienced a decline in QoL and physical and mental health during the pandemic. The pandemic also exacerbated socioeconomic and MH disparities, as noted by Lee and Singh [15], and increased the inequality gap by disproportionately impacting the most vulnerable groups of the older population [16].

Older adults did not experience the same impact on all health issues; memory problems, stomach problems, and feeling down specifically increased [17]. Peters et al. [18] demonstrated that SRH declined in individuals who underwent COVID-19 testing. By analyzing SHARE data, Silva et al. [19] revealed that individuals confined to their homes during the pandemic were more likely to have sleep difficulties and one or more chronic conditions than those not confined. The pandemic lockdown has led to increased unhealthy behaviours, especially among men, those lacking regular interaction with family and friends, and individuals with impaired overall health or chronic illnesses [20].

The pandemic also affected MH, with individuals living alone facing an elevated risk of severe psychological distress [21]. Lee [22] argued that “loneliness emerged as a significant predictor for the general SRH ratings,” while Zaninotto et al. [23] offered evidence of a general decline in various MH indicators. The social isolation measures have exacerbated MH issues such as depression, anxiety, and loneliness, especially among older adults [24]. According to Noguchi et al. [25], the isolation experienced by older adults during the COVID-19 crisis might be associated with their cognitive decline. Failing to address healthcare needs has been associated with a higher chance of experiencing a decline in health-related QoL and an increased likelihood of reporting a decrease in SRH [26]. Several other studies have shown that older adults with unmet healthcare needs face an increased risk of mortality and morbidity [27, 28], while others have underscored the marginalization of their healthcare, especially in countries implementing mitigation strategies [29]. Using Survey of Health, Ageing and Retirement in Europe (SHARE) data, Smolić et al. [30] argued that “those aged 60 or older, with limited healthcare access during the COVID-19 pandemic, were more likely to report worsened health status.” Similarly, Tur-Sinai et al. [31] confirmed a correlation between having medical appointments forgone, postponed, or denied and a higher probability of experiencing SRH deterioration during the pandemic.

In response to the lack of research enhancing our insight into health status changes among older Europeans amid the COVID-19 pandemic, we employ data from the SHARE study [32]. Our main objective is to investigate factors contributing to the decline in SRH of people aged 50 and above between 2020 and 2021. We rely on the fact that the pandemic disproportionately affected this population group [33, 34], making them a crucial focus for researchers. The specific objective of this study is to gain a more comprehensive insight into the impact of unmet healthcare needs along with COVID-19 affectedness on SRH deterioration during the COVID-19 health crisis. Through the presentation of our findings, this paper introduces the policy measures that might be useful in preserving the health status of vulnerable population groups in future health emergencies.

## Methods

### Sample

We use SHARE Corona Survey (SCS) datasets from waves 8 and 9 of the SHARE study. SHARE, initiated in 2004, is a longitudinal, multidisciplinary, international study containing data collected in 28 European countries and Israel. The SHARE data on individuals and their surroundings are ideal for studying individual and population ageing and the impacts of various policies (e.g., health, social, economic, and environmental) throughout their lives. Respondents in SHARE are drawn from households with at least one individual aged 50+ at the time of sampling, residing permanently in the respective SHARE country. Partners of eligible respondents, irrespective of age, are also eligible for the interview. Those in prison, hospitalized, living abroad at the time of the survey, unable to speak the respective SHARE country official language(s), or moved to an unknown address are considered ineligible [32].

To collect the data during the pandemic—which interrupted regular data collection via “computer-assisted personal interviews” (CAPI)—SHARE implemented two waves of SCS in 27 European countries and Israel. Data was gathered via “computer-assisted telephone interviews” (CATI) from June and August 2020 (1st SCS) and again 1 year later between June and August 2021 (2nd SCS), targeting only panel respondents [35, 36]. The SCS questionnaires addressed crucial aspects to evaluate the effects of the COVID-19 crisis on the 50+ population, such as effects on health, work-related and economic changes, and alterations in social networks [37]. Out of the 57,559 interviews in the 1st and 49,253 in the 2nd SCS, our working sample was restricted to 42,854 unique non-institutionalized persons aged 50+ who participated in both waves. In defining the sample, we excluded partners of main respondents younger than 50, interviews conducted by proxy respondents and missing values on all studied variables.

### Outcome Variable

As in “regular” SHARE waves, questions about SRH status have been asked in each SCS. The SRH is a commonly employed measure of objective health status, morbidity and mortality [38, 39]. Participants assessed the change in SRH in each SCS, comparing it with the SRH before the outbreak or with the SRH 3 months before the last wave in which they participated (wave 9). The questions used to assess the change in SRH were: “*If you compare your health with that before the outbreak of Corona, would you say your health has improved, worsened, or stayed about the same?*” in 1st SCS, and: “*If you compare your health now to 3 months ago, would you say your health has improved, stayed about the same, or worsened?*” in 2nd SCS. While there are slight variations, the questions enable us to evaluate changes in the SRH during the pandemic. Based on these questions, we have constructed a binary dependent variable that distinguishes between “SRH worsened” and “SRH improved or stayed about the same.”

### Predictors

Sociodemographic variables include sex, age divided into three groups (50–65, 65–79 and 80+), living arrangement (living alone or with others) and education based on the “International Standard Classification of Education” (ISCED-1997) [40]. To evaluate the economic situation, respondents were queried about facing challenges in making ends meet. Four original response options were dichotomized to differentiate individuals who experienced economic challenges during the pandemic from those who did not.

Our key predictors—denoting the subjective unmet healthcare during the pandemic—are all binary variables for healthcare forgone (when respondents refrained from healthcare utilization due to fear of coronavirus infection), healthcare postponed (when health professionals or facilities delayed scheduled medical treatments), and healthcare denied (instances where respondents requested a medical treatment appointment but did not receive it). Specifically, respondents could indicate the type of medical treatment they had forgone due to fear of infection, had postponed by medical staff or a facility, or been denied after requesting it, including treatment by a general practitioner, specialist (including a dentist), planned medical treatment (such as an operation), physiotherapy, psychotherapy, rehabilitation, or another type of medical treatment. Numerous studies have highlighted that challenges in meeting healthcare needs, particularly during the COVID-19 pandemic, adversely affected the health status of older adults [4, 41–43]. Furthermore, earlier studies confirmed a positive link between unmet healthcare needs and SRH decline [26], or poorer health status among those individuals reporting unmet healthcare needs [44]. The selection of these predictors has been influenced by the Andersen model of healthcare utilization [45], which explains that access to healthcare services and their utilization is modified by “predisposing” (e.g., age, sex, education), “enabling” (e.g., financial/economic situation) and “need” factors (e.g., number of chronic illnesses, presence of disability).

The affectedness by the COVID-19 pandemic is another predictor of interest. We created a variable indicating the impact of COVID-19, classified into three groups: not exposed individuals (lacking personal knowledge of anyone with symptoms, testing positive, being hospitalized, or died due to COVID-19), those mildly affected (having personal knowledge of someone with symptoms or tested positive, including the respondent), and those severely affected (having personal knowledge of someone, including the respondent, who was hospitalized or deceased due to COVID-19). A variable denoting wave 8 and wave 9 was added to examine the evolution of worsened SRH prevalence over time, i.e., between 2020 and 2021.

### Data Analyses

We begin our analysis by presenting the sample description by country from the 1st SCS, followed by a description of the outcome and predictors that changed between the 1st and 2nd SCS. Additionally, we conducted an exploratory data analysis to test the differences in the proportions of adults aged 50 and older with and without worsened SRH by predictors across both waves. The association between worsened SRH and our key predictors was explored by applying logistic mixed-effects regression analysis. The variable “wave” was implemented as a random slope, and the variable “country” served as a level-2 predictor. In our study, “wave” reflects changes between the two waves, while “country” captures variations across different countries. The model with a random slope for wave implies that the between-country variance depends on the wave in which the data were collected. We presented three distinct models—one for the entire sample and two additional models to examine differences in worsened SRH based on sex and age. Furthermore, predicted probabilities were computed to illustrate how changes of unmet healthcare variables and COVID-19 exposure affect the probability of worsened SRH. All analyses were carried out using STATA statistical software, version 18. In addition, this paper utilized ChatGPT (OpenAI, 2023, https://chat.openai.com/) for assistance with grammar checks.

## Results

The descriptives for sociodemographic variables by country are presented in Table 1. We only showed data for the 1st SCS as these variables remained relatively stable in both SCSs. Table 2 illustrates the sample characteristics that changed over time, such as perceived worsened SRH, economic situation, unmet healthcare and COVID-19 exposure.

**TABLE 1:** Sample description by country in the 1st SHARE Corona Survey (wave 8), % (EU-26, Israel, and Switzerland. 2020 and 2021).

	*N*	Age 50–64	Age 65–79	Age 80+	Female	Living alone	Low education	Medium education	High education
Austria	1,954	18.6	59.2	22.2	61.5	34.6	20.4	50.5	29.1
Germany	1,896	32.2	53.3	14.6	53.9	21.9	8.0	57.4	34.5
Sweden	888	15.3	65.5	19.1	56.0	27.1	27.0	32.4	40.5
Netherlands	652	22.5	65.5	12.0	54.4	27.0	37.9	27.5	34.7
Spain	1,370	19.8	58.5	21.7	59.1	18.8	77.3	11.2	11.5
Italy	2,910	28.6	54.5	16.9	56.5	15.9	65.4	25.6	9.1
France	1,643	25.6	57.3	17.1	59.5	32.1	31.0	39.4	29.5
Denmark	1,438	33.3	54.9	11.8	56.5	25.0	12.4	37.2	50.4
Greece	3,113	29.0	52.3	18.7	57.7	21.2	49.5	29.8	20.6
Switzerland	1,584	21.1	60.7	18.2	54.5	27.0	16.0	63.9	20.1
Belgium	3,097	32.9	52.5	14.6	56.7	28.2	31.8	28.9	39.3
Israel	926	14.0	64.4	21.6	58.1	22.5	26.7	29.9	43.4
Czechia	1,862	17.5	68.5	14.0	64.8	32.2	31.5	51.8	16.6
Poland	2,440	41.0	50.9	8.1	57.9	15.1	21.8	64.1	14.1
Luxembourg	702	37.2	53.7	9.1	54.8	19.5	36.6	38.0	25.4
Hungary	718	23.1	64.5	12.4	63.2	25.1	22.4	57.4	20.2
Portugal	856	27.3	60.0	12.6	58.4	16.0	77.2	10.2	12.6
Slovenia	2,618	27.2	58.2	14.7	59.9	19.2	27.1	54.9	18.0
Estonia	3,610	28.8	50.4	20.9	63.8	33.6	19.4	54.2	26.5
Croatia	1,623	36.5	53.8	9.7	57.4	16.8	30.4	52.7	16.9
Lithuania	1,150	42.4	41.4	16.2	64.1	31.0	15.6	44.9	39.6
Bulgaria	537	35.4	52.7	11.9	63.3	29.4	25.7	56.8	17.5
Cyprus	469	26.0	55.0	19.0	57.1	15.4	49.5	31.8	18.8
Finland	1,201	37.1	51.0	11.9	55.9	22.2	25.6	33.5	41.0
Latvia	855	35.9	48.1	16.0	64.7	32.0	13.9	58.1	28.0
Malta	609	35.8	55.0	9.2	59.6	13.0	41.5	50.7	7.7
Romania	1,330	43.2	47.7	9.2	58.3	15.0	45.3	48.1	6.5
Slovakia	803	54.0	40.7	5.2	55.5	18.6	10.2	82.6	7.2
Total	42,854	29.8	54.9	15.3	58.8	23.9	32.0	43.8	24.2

Note: unweighted data. Source: SHARE Wave 8 COVID-19 Survey release 8.0.0., and author’s calculations.

**TABLE 2:** Description of outcome and predictors that changed between 1st (wave 8) and 2nd SHARE Corona Survey (wave 9) by country, % (EU-26, Israel, and Switzerland. 2020 and 2021).

Country	1st SHARE corona survey (wave 8, 2020)						2nd SHARE corona survey (wave 9, 2021)					
	SRH worsened	Economic difficulties	Forwent healthcare	Postponed healthcare	Denied healthcare	Severe COVID	SRH worsened	Economic difficulties	Forwent healthcare	Postponed healthcare	Denied healthcare	Severe COVID
Austria	10.1	9.6	13.7	27.8	4.4	6.6	12.8	7.0	8.5	11.5	2.4	22.2
Germany	8.5	8.8	17.6	19.1	3.0	3.6	12.8	7.2	11.2	8.6	2.5	12.1
Sweden	7.5	8.1	16.9	20.5	4.5	9.8	8.1	7.1	14.4	12.4	3.7	16.1
Netherlands	6.4	4.8	9.0	36.3	4.4	12.0	8.7	3.8	6.6	16.7	2.6	18.1
Spain	9.3	22.9	4.5	32.2	4.4	13.7	13.7	19.2	1.9	13.4	3.7	19.3
Italy	8.6	43.1	15.7	27.9	6.1	10.3	12.7	41.0	10.8	13.9	6.2	18.8
France	11.0	16.1	10.6	35.8	10.3	7.2	10.8	17.7	9.2	13.5	10.4	14.4
Denmark	5.4	4.7	12.2	31.1	4.3	5.0	4.7	3.4	4.9	11.7	3.8	5.8
Greece	6.9	89.1	17.7	11.7	4.0	0.2	9.7	89.3	10.6	7.8	4.2	18.1
Switzerland	6.8	9.5	12.6	29.5	2.8	9.3	10.3	8.9	4.5	10.4	1.8	22.3
Belgium	9.3	14.9	14.1	36.8	7.0	16.1	12.5	14.4	11.9	18.6	3.8	22.6
Israel	12.1	34.9	22.7	26.9	5.5	5.7	22.8	24.0	14.6	11.1	3.8	14.9
Czechia	8.0	8.5	17.7	38.1	2.0	0.5	13.6	7.7	9.6	20.0	1.7	17.7
Poland	10.5	45.4	10.2	29.4	7.3	1.4	19.5	39.1	6.8	14.3	6.5	18.0
Luxembourg	8.5	7.8	23.5	52.0	7.3	10.5	12.3	7.7	11.4	18.9	4.4	24.1
Hungary	5.3	68.9	9.1	24.4	3.8	0.8	12.3	71.0	16.6	26.3	10.4	16.7
Portugal	12.5	49.5	10.9	50.1	7.9	4.7	17.2	46.6	8.3	30.6	8.9	18.7
Slovenia	5.0	45.1	4.9	34.5	2.9	0.4	11.7	36.2	4.3	16.8	3.9	9.9
Estonia	6.0	32.4	11.1	25.2	7.3	1.2	15.1	29.5	6.0	8.2	4.9	8.5
Croatia	9.6	59.0	9.1	24.6	3.2	1.0	16.7	53.0	7.8	9.7	4.3	12.6
Lithuania	13.7	40.0	13.2	29.0	11.7	0.6	20.4	36.2	11.2	10.1	8.5	16.9
Bulgaria	6.9	67.8	10.8	1.5	0.7	1.5	22.7	54.0	4.3	3.9	2.6	30.5
Cyprus	7.5	51.8	12.2	19.6	4.3	1.9	13.4	46.1	6.6	3.8	1.3	11.5
Finland	9.2	14.2	8.4	21.2	4.7	1.5	8.6	14.9	6.7	14.2	5.3	3.2
Latvia	6.8	49.9	14.2	15.7	7.4	0.2	18.9	53.7	7.4	3.5	3.2	8.7
Malta	8.5	36.6	11.8	38.3	2.8	4.1	16.1	22.0	12.2	19.5	2.6	9.0
Romania	7.6	67.2	4.7	7.1	4.0	2.9	15.2	69.7	1.4	2.8	0.8	11.5
Slovakia	6.2	50.9	14.6	21.0	5.4	0.1	14.3	49.6	13.0	22.2	6.4	10.7
Total	8.3	34.5	12.6	27.4	5.3	4.9	13.4	32.0	8.4	13.0	4.5	15.4

Note: unweighted data. Source: SHARE Wave 8 COVID-19 Survey release 8.0.0., Wave 9 COVID-19 Survey release 8.0.0., and authors’ calculations.

The mean age in the 1st SCS was 69.9 years, with 54.9% of the sample falling between the ages of 65 and 79. Approximately 24% of older adults lived alone, almost 59% of the sample were females, and 24.2% had a high educational level. Descriptives for the variables that changed between the two waves are presented in Table 2. We observe an increase in the prevalence of worsened SRH from 8.3% to 13.4%, as well as a rise in the proportion of older adults severely affected by COVID-19, from 4.9% to 15.4%. On one hand, the prevalence of worsened SRH in the 1st SCS was highest in Lithuania, Portugal, and Israel. In the 2nd SCS, the highest prevalence was observed in Israel, Bulgaria, and Lithuania. On the other hand, the countries with the largest increases in prevalence between the two surveys were Bulgaria, Latvia, and Israel, with increases of 15.8, 12.1, and 10.7 percentage points (pp.), respectively. Countries that exhibited a decrease in the proportion of older adults with worsened SRH were Denmark, Finland, and France (see Supplementary Figure S1). However, France showed a proportion of 11.0% in wave 8% and 10.8% in wave 9, indicating relative stability. All countries showed an increase in the share of older adults severely affected by COVID-19. The most dramatic increase was noted in Bulgaria (29 pp.), followed by Greece (17.9 pp.) and Czechia (17.2 pp.). This increase could be partially explained by an escalation of the pandemic after relative success in controlling the spread of the coronavirus during its initial wave. The following three countries with the largest increases were Poland, Lithuania, and Hungary, all significantly affected by the virus spread at the end of 2020 and the beginning of 2021 [46, 47].

Additionally, there has been a slight decline in the share of those with economic difficulties. More significantly, three dimensions of unmet healthcare needs during the pandemic exhibited notable changes. Specifically, the prevalence of postponed healthcare dropped from 27.4% to 13%, healthcare forgone decreased from 12.6% to 8.4% and denied healthcare decreased from 5.3% to 4.5%. We further examined the variation in the percentage of older adults experiencing worsened SRH and those with unchanged or improved SRH based on predictors of unmet healthcare during the pandemic (refer to Supplementary Figures S2, S3). In almost all countries within the sample and for each wave, we identified relatively larger proportions of older adults reporting SRH deterioration among those who had their medical treatments postponed or denied and who refrained from seeking medical care for fear of infection. The difference between the categories of outcome variable in 1st SCS was 10 pp. in case of healthcare forgone, 12.1 pp. for postponed, and 7.1 pp. for denied medical care, but this dropped to 4.3 pp., 5.1 pp. and 4.7 pp. in the 2nd SCS, respectively.

Our exploratory data analysis is presented in Table 3, which shows the differences in the proportions of adults aged 50 and older whose SRH either worsened or did not worsen (remained unchanged or improved) by sex, age, living arrangement, education, and other predictors across both waves of the SCS. We conclude that females were more likely to report deteriorated SRH, as were the oldest old (80+). Lone living, low education, and economic difficulties were significantly associated with greater odds of reporting deteriorated SRH. In addition, those who experienced unmet healthcare during the pandemic had greater odds of worsened SRH. The prevalence of worsened SRH was also higher among individuals who faced severe exposure to the pandemic.

**TABLE 3:** Sample characteristics (proportions) for the 1st and 2nd SHARE Corona Survey (waves 8 and 9) (EU-26, Israel, and Switzerland. 2020 and 2021).

Predictor	Wave 8		Wave 9	
	Not worsened SRH	Worsened SRH	Not worsened SRH	Worsened SRH
Female	0.58	0.66 ***	0.58	0.64 ***
Age				
50–64	0.30	0.24 ***	0.27	0.20 ***
65–79	0.55	0.53 **	0.57	0.52 ***
80+	0.15	0.23 ***	0.16	0.28 ***
Living alone	0.23	0.31 ***	0.24	0.32 ***
Education				
Low	0.32	0.38 ***	0.31	0.40 ***
Medium	0.44	0.40 ***	0.44	0.41 ***
High	0.24	0.23 **	0.25	0.19 ***
Economic difficulties	0.34	0.44 ***	0.30	0.43 ***
Forwent healthcare	0.12	0.22 ***	0.08	0.12 ***
Postponed healthcare	0.26	0.39 ***	0.12	0.17 ***
Denied healthcare	0.05	0.12 ***	0.04	0.09 ***
Affected by COVID				
None	0.85	0.79 ***	0.53	0.51 **
Mild	0.10	0.14 ***	0.32	0.31 **
Severe	0.05	0.07 ***	0.15	0.18 ***
*N*	39,317	3,537	37,095	5,759

Source: SHARE Wave 8 COVID-19 Survey release 8.0.0., Wave 9 COVID-19 Survey release 8.0.0., and authors’ calculations. **p < 0.05, ***p < 0.01. Note: A chi-square test was employed to examine the association between predictors and outcome.

The results of logistic mixed-effects regression analyses (Table 4) were displayed for the overall sample and then separated by sex and three age categories. The model included fixed effects for predictors and a random intercept for country to account for baseline differences across countries. This approach enabled us to better understand the association between unmet healthcare during the pandemic and worsened SRH across specific population segments. We presented and interpreted our findings, focusing on odds ratios (OR) and predicted probabilities of the outcome for significant predictors between the 1st and 2nd SCS. In our study, the odds ratios demonstrate how the likelihood of worsened SRH changes with a one-unit increase in the predictor variable, holding all other predictors constant. Predicted probabilities show how changes in a predictor affect the probability of an outcome, in our case worsened SRH between two waves.

**TABLE 4:** Logistic mixed model regressions of worsened self-reported health (EU-26, Israel, and Switzerland. 2020 and 2021).

	All			Sex						Age								
	OR	[95% CI]		Female			Male			50–64			65–79			80+		
				OR	[95% CI]		OR	[95% CI]		OR	[95% CI]		OR	[95% CI]		OR	[95% CI]	
Female (ref. male)	1.20 ***	1.15	1.26							1.20 ***	1.09	1.33	1.21 ***	1.13	1.29	1.21 ***	1.10	1.34
Age (ref. 50–64)																		
65–79	1.27 ***	1.20	1.35	1.26 ***	1.18	1.36	1.29 ***	1.17	1.42									
80+	2.31 ***	2.15	2.48	2.26 ***	2.07	2.47	2.42 ***	2.16	2.72									
Living alone (ref. living with others)	1.17 ***	1.11	1.23	1.16 ***	1.09	1.23	1.23 ***	1.11	1.35	1.27 ***	1.12	1.44	1.19 ***	1.11	1.27	1.10 *	1.00	1.21
Education (ref. low)																		
Medium	0.83 ***	0.78	0.87	0.82 ***	0.77	0.88	0.84 ***	0.76	0.92	0.86 **	0.76	0.98	0.82 ***	0.76	0.88	0.82 ***	0.73	0.92
High	0.79 ***	0.74	0.84	0.77 ***	0.71	0.83	0.83 ***	0.74	0.92	0.82 **	0.71	0.96	0.74 ***	0.67	0.81	0.91	0.80	1.03
Economic difficulties	1.79 ***	1.71	1.89	1.77 ***	1.67	1.90	1.81 ***	1.66	1.97	1.88 ***	1.70	2.11	1.74 ***	1.62	1.87	1.76 ***	1.58	1.97
Forwent healthcare	1.57 ***	1.48	1.68	1.56 ***	1.45	1.69	1.60 ***	1.42	1.80	1.96 ***	1.74	2.27	1.48 ***	1.36	1.62	1.40 ***	1.22	1.62
Postponed healthcare	1.40 ***	1.33	1.48	1.43 ***	1.33	1.53	1.36 ***	1.23	1.49	1.44 ***	1.28	1.61	1.40 ***	1.30	1.51	1.34 ***	1.19	1.51
Denied healthcare	2.03 ***	1.89	2.23	2.03 ***	1.85	2.27	2.05 ***	1.79	2.38	2.20 ***	1.90	2.62	1.99 ***	1.80	2.25	1.89 ***	1.57	2.30
Affected by COVID (ref. none)																		
Mild COVID	1.19 ***	1.10	1.23	1.21 ***	1.10	1.27	1.17 ***	1.05	1.27	1.43 ***	1.22	1.55	1.18 ***	1.08	1.26	0.99	0.86	1.11
Severe COVID	1.36 ***	1.25	1.44	1.35 ***	1.22	1.46	1.38 ***	1.22	1.54	1.55 ***	1.29	1.75	1.32 ***	1.19	1.44	1.32 ***	1.15	1.54
SHARE wave 9	1.76 ***	1.55	2.01	1.74 ***	1.49	2.00	1.81 ***	1.59	2.05	1.61 ***	1.33	1.90	1.69 ***	1.50	1.91	2.07 ***	1.77	2.51
Constant	0.04 ***	0.04	0.05	0.05 ***	0.04	0.06	0.04 ***	0.03	0.04	0.03 ***	0.03	0.04	0.06 ***	0.05	0.06	0.09 ***	0.07	0.10
Fixed effects																		
	0.57 (0.07)			0.55 (0.07)			0.59 (0.07)			0.46 (0.09)			0.53 (0.06)			0.75 (0.09)		
Random effects parameters																		
Intercept	0.23 (0.08)			0.29 (0.10)			0.06 (0.03)			0.35 (0.15)			0.13 (0.06)			0.35 (0.16)		
Slope	0.10 (0.03)			0.12 (0.04)			0.11 (0.06)			0.13 (0.06)			0.07 (0.03)			0.12 (0.05)		
Covariance	−0.13 (0.05)			−0.16 (0.06)			−0.06 (0.04)			−0.19 (0.09)			−0.07 (0.04)			−0.18 (0.09)		
*N*	85.708			50.364			35.344			23.802			47.698			14.208		

Source: SHARE Wave 8 COVID-19 Survey release 8.0.0. Wave 9 COVID-19 Survey release 8.0.0., and authors’ calculations. *p < 0.1. **p < 0.05. ***p < 0.01. Notes: SE in parentheses. OR, odds ratio. The random intercept variance for the full sample for country is 0.23 (SE = 0.08). The random slope for variance for the dummy variable indicating the SHARE wave is 0.10 (SE = 0.03) and the covariance (correlation) between the random intercept and slope is −0.13. The fixed effect coefficient is the average effect of dummy variable indicating the SHARE wave across all countries β = 0.57. SE = 0.07. z = 8.48. *p* < 0.001.

The initial finding from the full sample indicates that older Europeans had higher odds of reporting SRH worsening in the 2nd SCS (wave 9) than in the 1st SCS (wave 8). Factors such as female sex (OR 1.20), belonging to older age groups (65–79 and 80+, with OR 1.27 and 2.31, respectively), and living alone (OR 1.17) were found as significant predictors of deteriorating SRH. The likelihood of worsened SRH was lower for individuals with medium (OR 0.83) or high educational levels (OR 0.79). Conversely, facing economic difficulties significantly increased the odds of worsened SRH (OR 1.79). For our key variables—three aspects of unmet healthcare during the pandemic—we observed statistically significant associations between healthcare forgone (OR 1.57), postponed (OR 1.40), and denied (OR 2.03) with worsened SRH among older Europeans. Lastly, the seriousness of the COVID-19 impact increased the likelihood of worsened SRH. For instance, respondents severely exposed to COVID-19 exhibited 36% higher odds of worsened SRH compared to those not exposed at all. We further observe that the fixed effect of wave on worsened SRH was significant [β = 0.57, SE = 0.07, z = 8.48, *p* < 0.001], indicating that, on average, an increase in wave—from SHARE wave 8 to wave 9—was associated with higher odds of worsened SRH among older adults. The random intercept variance of 0.23 (SE = 0.08) indicates variability in the baseline levels of the worsened SRH across countries. When examining the effect of wave by country, it varied significantly (variance = 0.10, SE = 0.03), suggesting that the impact of wave on the outcome variable differs across SHARE countries. This indicates that country-specific factors influenced the worsening of SRH between the two waves of data collection. The covariance (or correlation) between the random intercept and slope was −0.13, indicating a negative relationship between countries’ baseline levels and the strength of the wave effect. We could also argue this in a way that in countries with higher baseline probabilities of the outcome (worsened SRH in wave 8 compared to pre-pandemic levels), the effect of the “wave” variable on the outcome tends to be weaker. In other words, the impact of the predictor “wave” decreases as the baseline level of the outcome increases across countries.

While separate regressions for males and females revealed fairly similar odds of reporting SRH deterioration, a slightly different pattern emerges from the regressions by age spline. Occupationally active adults (50–64 age group), especially those among them who were living alone or facing economic difficulties, demonstrated the highest odds of worsened SRH compared with their older counterparts. Furthermore, experiencing unmet healthcare during the pandemic had the most significant effect on the likelihood of worsened SRH for individuals in the 50–64 age group. The severe affectedness by the COVID-19 increased the odds of reporting worsened SRH, particularly for those in the 50–64 age group (OR = 1.55, 95% CI [1.29, 1.75], *p* < 0.001) compared to 65–79 (OR = 1.32, 95% CI [1.19, 1.44], *p* < 0.001) and 80+ (OR = 1.32, 95% CI [1.15, 1.54], *p* < 0.001) age groups. Among the oldest old (80+) we observe the highest wave effects on the odds of reporting worsened SRH (OR = 2.07, 95% CI [1.77, 2.51], *p* < 0.001) compared to the other two age groups: 50–64 (OR = 1.61, 95% CI [1.33, 1.90], *p* < 0.001) and 65–79 (OR = 1.69, 95% CI [1.50, 1.91], *p* < 0.001). For the entire sample, we found that the fixed effect of wave on the outcome was significant within the restricted samples by sex and age groups. The covariances between the random intercept and random slope in each logistic mixed-effects model were negative, indicating once again that the impact of the “wave” variable decreases as the baseline level of the outcome increases across countries. However, the correlation between the outcome and wave was more negative for females compared to males and for the 50–64 age group compared to the two older groups. According to theory, a more negative covariance suggests a greater reduction in the effect of the “wave” variable as the baseline outcome level rises, particularly for those who reported worsened SRH in wave 8.

**FIGURE 1:**
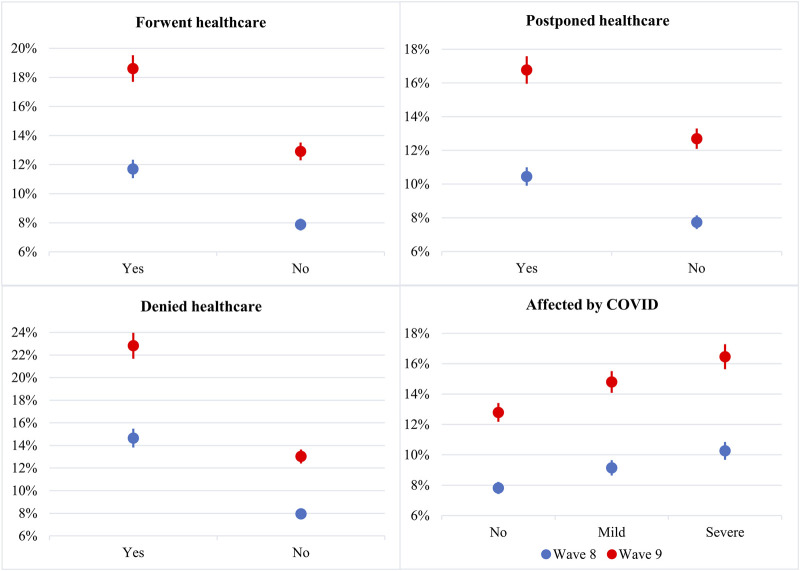
Predicted probabilities of worsened self-reported health by predictors and wave, showing changes between the 1st and 2nd SHARE Corona Surveys (EU-26, Israel, and Switzerland. 2020 and 2021). Note: the lines represent 95% confidence intervals.

When examining the predicted probabilities for the SRH decline between two waves, as illustrated in Figure 1, it is apparent that deteriorated SRH was generally more common in the 2nd SCS (wave 9). This trend was particularly pronounced for individuals who had experienced unmet healthcare during the pandemic, resulting in a more robust increase in reported worsened SRH. Regarding COVID-19 exposure, the pattern remained consistent: worsened SRH was more likely in the 2nd SCS for every category of the “COVID-19 exposure” variable.

## Discussion

Many researchers have raised concerns about the negative impact of unmet healthcare needs [12, 17, 20, 24] and COVID-19 exposure and related restrictions [9, 13, 48, 49] on older adults’ physical and mental health during the pandemic. As a contribution to the existing pool of studies, this paper provides deeper insight into the interplay of unmet healthcare needs during the COVID-19 pandemic and changes in SRH of the population aged 50 and above in the European context. Furthermore, we shed light on the impact of exposure to the effects of the pandemic on the decline of SRH. Equally importantly, this study is based on a unique, harmonized, and largest panel database on the lives of older Europeans during the COVID-19 pandemic.

The main problem addressed in this study is understanding whether restrictions on healthcare provision and disruptions in accessing healthcare—frequently observed during the pandemic [50]—contributed to the decline in SRH of older adults. When establishing the link between the context of the pandemic and the changes in SRH of older adults, we studied three non-standard dimensions of barriers to accessing healthcare during the pandemic: healthcare forgone due to fear of infection, postponed scheduled medical treatments and denied medical care [4]. We firmly embraced the conceptualization from “Andersen’s behavioural model of health services use” which argues that access to healthcare services and their utilization is influenced by predisposing, enabling, and need factors, all of which may have implications for an individual’s health outcomes [45, 51].

Our analyses showed that 8.3% of older adults in 27 European countries and Israel reported a deterioration in SRH around 3–4 months after the outbreak compared with their health before the pandemic. However, approximately 1 year later, this percentage increased to 13.4% when respondents were asked to compare their health status at the moment of the 2nd SCS interview with that from 3 months earlier (e.g., around March-May 2021). On the other hand, there was an evident reduction in each of the three dimensions of unmet healthcare between the two waves of SCS, with the most significant decline in the share of older adults facing the postponement of their scheduled medical treatments, from 27.4% to 13%. One could argue that this finding is supported by the pattern of the sharp disruption in healthcare provision in the initial stages of the pandemic [41, 52–54] and the restoration of healthcare services in its later stages, especially after vaccination rollout across Europe [55, 56]. We could also note that previously “COVID-19-focused healthcare systems” began to deal with backlogs in specialist and hospital care in the subsequent stages of the pandemic [57, 58], which probably contributed to a decrease in overall reported unmet healthcare.

Several other equally important findings emerged from this study. First, females were more likely to experience health deterioration, as were individuals in higher age groups, and this does not differ from the earlier findings from the pandemic times [13] as well before the pandemic [59]. Second, older adults who lived alone throughout the pandemic—nearly 24% of those aged 50 and above—experienced more adverse effects on their health status. Even though the impact of lone living on SRH deterioration was not very strong, it was significant and in line with the findings presented in other studies conducted during the pandemic. For instance, older adults who lived alone during the pandemic were more susceptible to MH issues [23, 60], while the pandemic further exacerbated vulnerabilities for those already limited in activities of daily living [61]. Finally, we have shown that a less favourable economic situation, considered an enabling factor for healthcare services utilization, negatively impacted the SRH of older adults during the pandemic. It has been shown elsewhere that “worse financial situation and lowered income were associated with treatment cancellation and accessible care” for elderly during the outbreak in the United Kingdom [62] and worsening MH [63].

Conversely, economic vulnerability is found to be notably higher among individuals who had poor health—and thus more vulnerable—before the outbreak [41]. Additional data analyses for specific subgroups (e.g., females or the oldest-old) exhibit comparable findings as from the whole sample. It should be noted, however, that some effects were more robust for specific age groups. On the one side, the impact of healthcare forgone or denied on worsening SRH was more robust for younger older adults (aged 50–64), while on the other side, the wave-time effect on SRH deterioration was strongest for those aged 80+. Finally, we demonstrated that mild and severe exposure to the pandemic significantly impacted the decline of SRH in older adults. In addition, we demonstrated the effect of pandemic exposure on the probability of SRH deterioration was more evident in wave 9 (2nd SCS), which is coherent with the pandemic expansion in late 2020 or early 2021 with more contagious and resistant variants of the coronavirus [64].

A deeper understanding of two dimensions of the negative impact of the pandemic on the health of older adults—unmet healthcare needs and COVID-19 exposure—is particularly important for understanding the long-term health impacts of the pandemic. Implementing early intervention measures and regularly monitoring health status can facilitate identifying and managing deteriorating health outcomes among older adults. This may entail proactive outreach, telehealth services, and community-based interventions tailored to support at-risk people—the oldest old, females, those living alone and in poor economic conditions and those who have been exposed to the pandemic the most. The increase in postponed and denied healthcare between the 1st and 2nd SCS in certain Central and Eastern European countries, such as Slovakia and Hungary, highlights significant geographical disparities in healthcare access during the COVID-19 pandemic. This may be due to the design of their healthcare systems, which are more hospital-centered and have significantly lower densities of health personnel compared to those in Western, Northern, or Southern Europe. Policymakers should prioritize reallocating resources to shift from cure-focused healthcare systems to those that emphasize prevention, health promotion, and more flexible and efficient methods of service provision. Strengthening healthcare systems to withstand crises and uphold essential services is crucial. Investing in adequate infrastructure, workforce capacity, and adaptability to changing demands can help mitigate disruptions in healthcare provision during emergencies. Moreover, gaining more precise insights into the unintended consequences of the pandemic on healthcare access will enhance the resilience of healthcare systems in the future. This can be achieved by leveraging available data and research findings to make informed decisions, address healthcare disparities, and allocate resources effectively. Our empirical findings clearly indicate that health interventions should be tailored—potentially made more intense, comprehensive, or specifically adapted—when targeting individuals or populations that have already experienced worsened SRH at baseline, in order to achieve the same effect as in groups who reported stable or improved health status.

### Strengths and Limitations

This study possesses numerous strengths. Unique data collected at two points throughout the pandemic, encompassing a large representative sample from 27 European countries and Israel and being one of the few panel studies providing insight into the impact of unmet healthcare during the pandemic on the health deterioration of older adults, are among them. However, it is important to note some limitations. It is possible that our regression model estimates may be somewhat biased due to not applying weights. Most variables used in the analyses were self-reported, which introduces the possibility of potential biases. Additionally, we employed unconventional predictors to assess the unmet healthcare needs of older adults, all of which are related to epidemic control decisions.

### Conclusion

This study delves into the multifaceted impacts of the COVID-19 pandemic on the health of older adults, focusing on unmet healthcare needs and exposure to the pandemic. By scrutinizing these dimensions, we explored long-term health effects on older Europeans aged 50 and above. Our findings underscore the critical importance of addressing barriers to healthcare access and understanding their association with SRH deterioration. Notably, while initial disruptions to healthcare provision were evident, subsequent efforts to restore services have been observed, albeit with varying impacts across age groups and socioeconomic statuses. Moreover, our analysis highlights sex disparities, the significance of social isolation, and the interplay between economic vulnerability and health outcomes. Moving forward, policymakers must prioritize proactive interventions, bolster healthcare systems’ resilience, and leverage data-driven insights to mitigate the enduring impact of health crises on older adults. Through concerted efforts, we can strive to build more resilient healthcare systems and safeguard the wellbeing of older adults facing future challenges.
