# Supplementary material for: The Impact of Unmet Healthcare Needs on the Perceived Health Status of Older Europeans During COVID-19
Source: Int J Public Health. 2024 Sep 30;69:1607336. doi: 10.3389/ijph.2024.1607336 (PMC11471687; doi:10.3389/ijph.2024.1607336)
Supplement: Supplementary file 1 [file DataSheet1.pdf]

The Impact of Unmet Healthcare Needs on the Perceived Health Status of Older Europeans During COVID-19

Supplementary Material

Figure S1. Proportion of older adults aged 50 and over with worsened self-reported health, 1st SHARE Corona Survey [wave 8 (2020)] and 2nd SHARE Corona Survey [wave 9 (2021)] (EU-26, Israel, and Switzerland. 2020 and 2021.

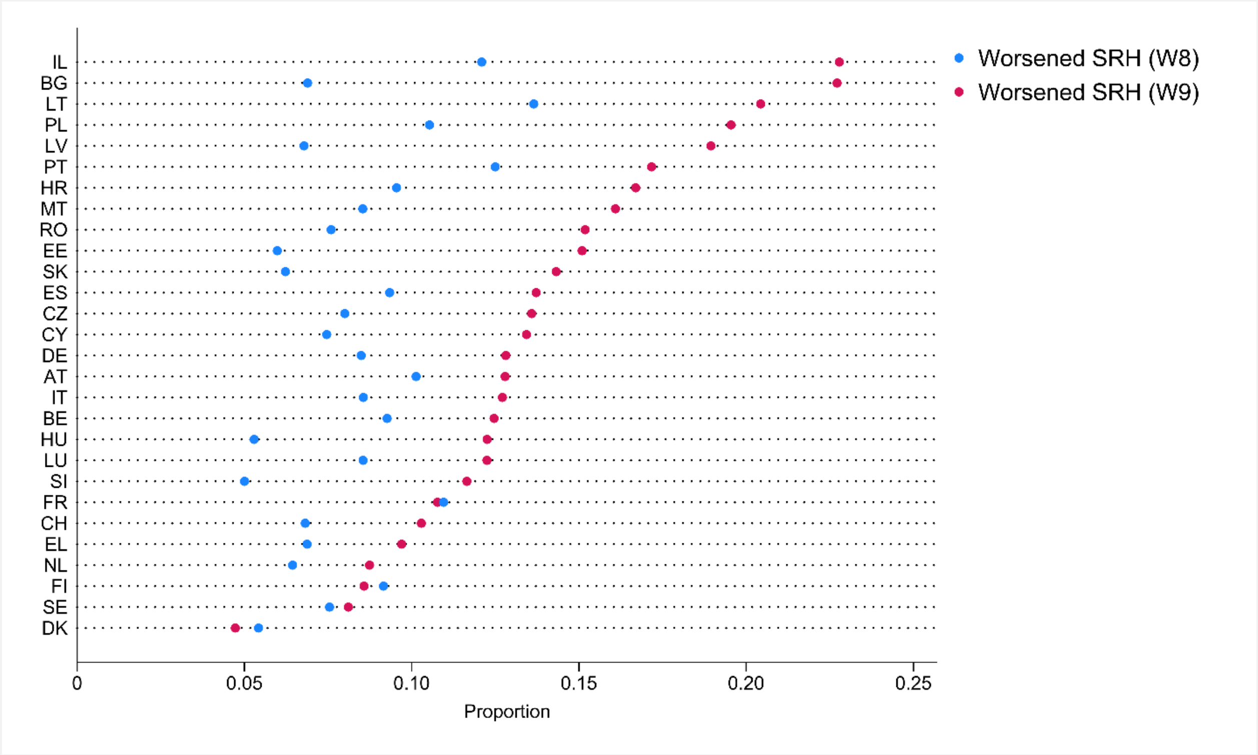

Source: SHARE Wave 8 COVID-19 Survey release 8.0.0., Wave 9 COVID-19 Survey release 8.0.0., and authors' calculations.

Figure S2. The difference in the percentage of adults aged 50 and older with worsened self-reported health and unchanged or improved self-reported health by healthcare forgone, postponed, denied, 1st SHARE Corona Survey, wave 8 (2020), (EU-26, Israel, and Switzerland. 2020).

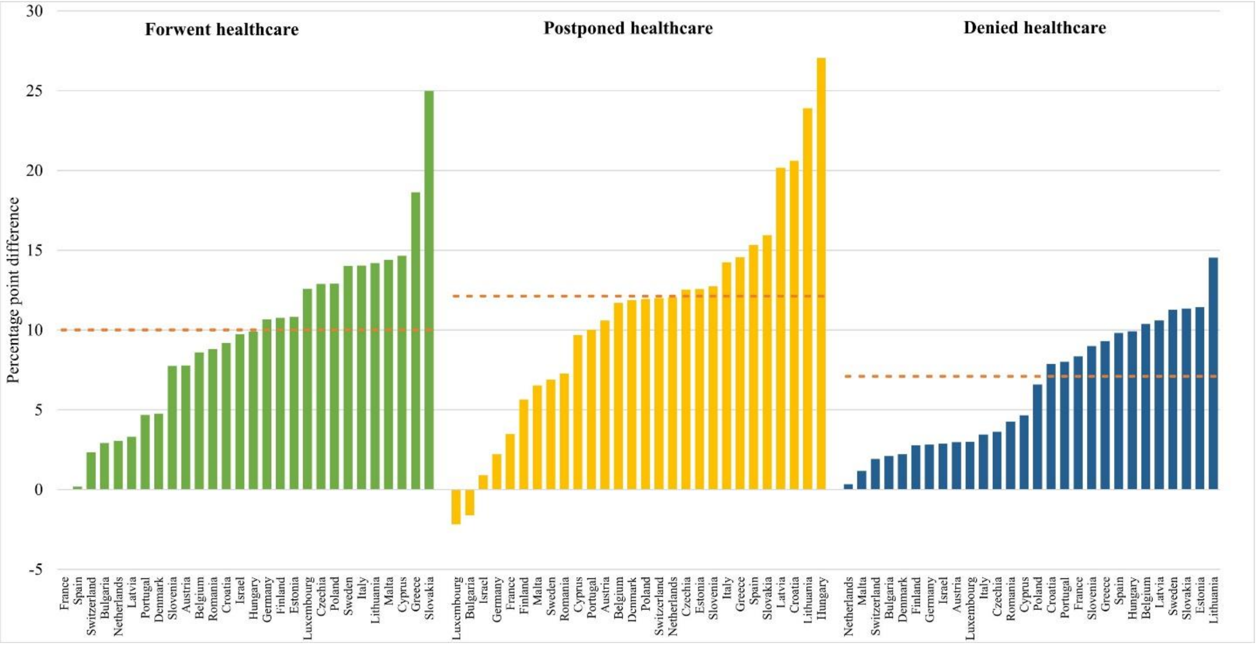

Note: unweighted data. Dashed lines represent the average difference. Source: SHARE Wave 8 COVID-19 Survey release 8.0.0., and authors’ calculations.

Figure S3. The difference in the percentage of adults aged 50 and older with worsened self-reported health and unchanged or improved self-reported health by healthcare forgone, postponed, denied, 2nd SHARE Corona Survey, wave 9 (2021), (EU-26, Israel, and Switzerland. 2021).

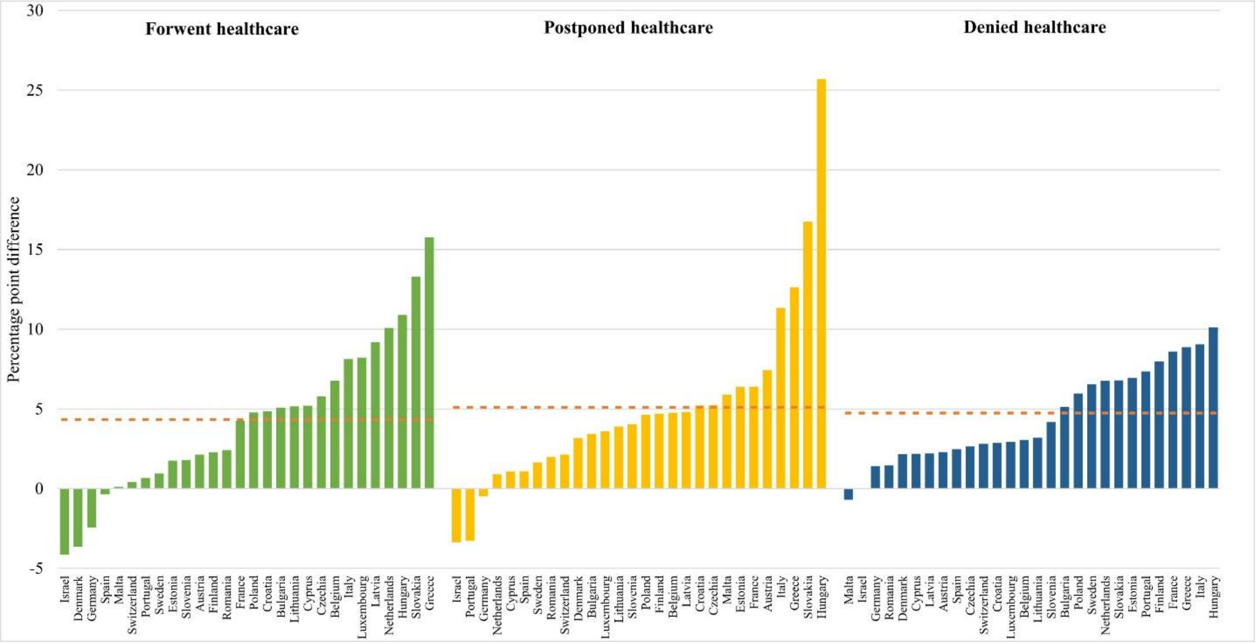

Note: unweighted data. Dashed lines represent the average difference. Source: SHARE Wave 9 COVID-19 Survey release 8.0.0., and authors' calculations.
